# Tumor Exosome Mimicking Nanoparticles for Tumor Combinatorial Chemo-Photothermal Therapy

**DOI:** 10.3389/fbioe.2020.01010

**Published:** 2020-08-31

**Authors:** Ran Tian, Zhaosong Wang, Ruifang Niu, Hanjie Wang, Weijiang Guan, Jin Chang

**Affiliations:** ^1^School of Life Sciences, Tianjin University, Tianjin, China; ^2^Tianjin Key Laboratory of Function and Application of Biological Macromolecular Structures, Tianjin Engineering Center of Micro-Nano Biomaterials and Detection-Treatment Technology, Tianjin, China; ^3^Public Laboratory, Tianjin Medical University Cancer Institute and Hospital, National Clinical Research Center for Cancer, Key Laboratory of Cancer Prevention and Therapy, Tianjin’s Clinical Research Center for Cancer, Tianjin, China; ^4^State Key Laboratory of Chemical Resource Engineering, College of Chemistry, Beijing University of Chemical Technology, Beijing, China

**Keywords:** tumor exosome, biomimetic, nanovehicles, breast cancer, combination therapy

## Abstract

The development of biomimetic nanoparticles with functionalities of natural biomaterial remains a major challenge in cancer combination therapy. Herein, we developed a tumor-cell-derived exosome-camouflaged porous silicon nanoparticles (E-MSNs) as a drug delivery system for co-loading ICG and DOX (ID@E-MSNs), achieving the synergistic effects of chemotherapy and photothermal therapy against breast cancer. Compared with ID@MSNs, the biomimetic nanoparticles ID@E-MSNs can be effectively taken up by the tumor cell and enhance tumor accumulation with the help of the exosome membrane. ID@E-MSNs also retain the photothermal effect of ICG and cytotoxicity of DOX. Under 808 nm near infrared irradiation, ICG can produce hyperthermia to collapse E-MSNs nanovehicles, accelerate drug release, and induce tumor ablation, achieving effective chemo-photothermal therapy. *In vivo* results of 4T1 tumor-bearing BALB/c mice showed that ID@E-MSNs could accumulate tumor tissue and inhibit the growth and metastasis of tumor. Thus, tumor exosome-biomimetic nanoparticles indicate a proof-of-concept as a promising drug delivery system for efficient cancer combination therapy.

## Introduction

Cancer with high mortality has become the leading cause of fatality worldwide (Bray et al., 2018), mainly due to the limited drug delivery system (Gelband et al., 2016). An ideal drug delivery achieves therapeutic efficacy in cancer with enhanced thermal target and long blood circulation (Maeda et al., 2013). In order to improve the capacity of targeting tumor tissues, nanoparticles have been surface-modified by peptides or chemical biomacromolecules (Cheng et al., 2015). However, nanoparticles as allogenic substances might be rapidly recognized and cleared away by the immune system (Salvati et al., 2013). Moreover, the targeting ligands are not valid for all types of tumors because of the complexity of tumors and heterogeneity of human beings (Zhao et al., 2013).

Biomimetic nanoparticles (Luk and Zhang, 2015; Tan et al., 2015; Zhen et al., 2019) are assembled by natural biomaterials such as cell membranes from cancer cells (Chen et al., 2016; Zhang et al., 2020), red blood cells (Gao et al., 2013; Piao et al., 2014), white blood cells (Parodi et al., 2013), platelets (Wei et al., 2016), and various synthetic nanoparticles, and this might be a promising strategy for anti-tumor drug delivery (Li et al., 2018). The biomimetic nanoparticles have displayed target-homing capacity, prolonged circulation, and good biocompatibility, in accordance with properties of cell membranes (Gao et al., 2016; Deng et al., 2018; He et al., 2018). Recently, exosomes as endogenous nanovesicles are secreted by various cells which have been developed as a novel drug delivery system (Batrakova and Kim, 2015; Vader et al., 2016). Moreover, numerous studies have reported that the exosomes 30–100 nm in diameter possess retention effects, and the membrane protein, target-homing and escaping phagocytosis (Kamerkar et al., 2017; Teng et al., 2017). Given all of these excellent characteristics, exosomes can be used as a drug delivery due to biocompatibility, low immunogenicity, and target-homing (Cheng et al., 2018). However, the exosomes used as a drug carrier are limited to the low drug-loading capacity (Yong et al., 2019), and porous silicon nanoparticles (MSNs) have been widely used for drug delivery owing to their excellent drug loading capacity (Wang et al., 2015). Therefore, it is desired to construct exosome-biomimetic nanoparticles with good biocompatibility and high drug loading for cancer therapy in present research.

The strategy of exosome-biomimetic nanoparticles with chemotherapy and photothermal therapy (Li J. et al., 2019; Cong et al., 2020; Guo et al., 2020; Li and Pu, 2020) could provide a novel approach for the combined treatment of tumors (Wan et al., 2018; Tang et al., 2019). As one of the commonly used photosensitizers, ICG is designed to exhibit PTT efficiency to induce the tumor cell apoptosis under 808 nm laser irradiation (Xin et al., 2017; Li X. et al., 2019). The utilization of ICG in combination with doxorubicin (DOX), a broad-spectrum chemotherapeutic drug, can improve anticancer effects (Ye et al., 2020; Wu et al., 2020). However, the hydrophobic characteristic of ICG and the toxicity of DOX *in vivo* limit its clinical applications (Zheng et al., 2013; Yan et al., 2016). Therefore, ICG and DOX can be co-loaded into exosome-biomimetic nanoparticles, which can improve drug stability, utilization, and effectivity so as to achieve the anti-tumor treatment.

**FIGURE 1:**
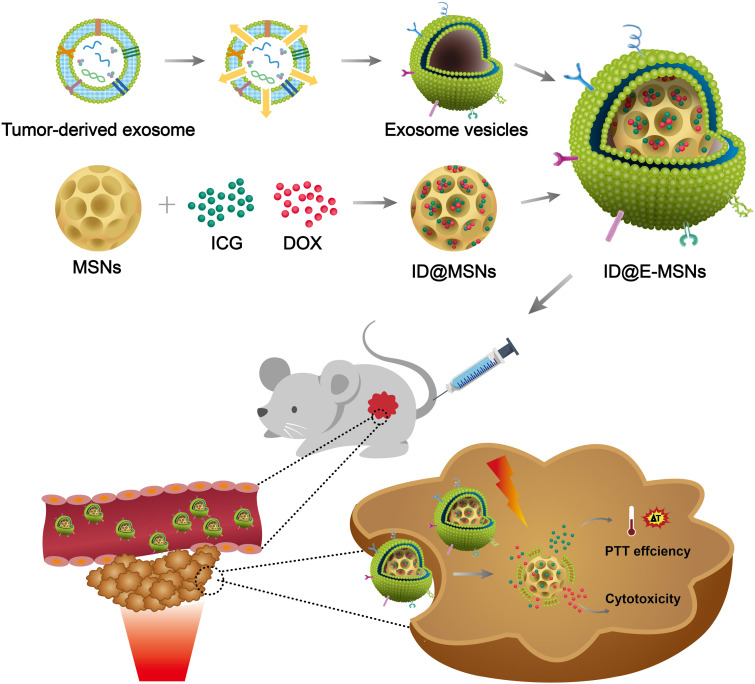
Schematic illustration of ID@E-MSNs nanovehicles for cancer-targeted chemo-photothermal therapy.

In this study, we developed a tumor-cell-derived exosome-camouflaged porous silicon nanoparticles (E-MSNs) by extrusion method (Pan et al., 2020) as a drug delivery system for co-loading ICG and DOX (ID@E-MSNs), to achieve targeted cancer combined therapy. Compared with ID@MSNs, the biomimetic nanoparticles ID@E-MSNs can be taken up by the tumor cell effectively, which could also enhance tumor accumulation owing to the characteristics of exosome membrane. Besides, ID@E-MSNs also retain the photothermal effect of ICG and cytotoxicity of DOX. Under 808 nm near infrared irradiation, ICG can produce hyperthermia to collapse nanovehicles, accelerate drug release, and induce tumor ablation, achieving effective chemo-photothermal therapy. *In vivo* results of 4T1 tumor-bearing BALB/c mice, it showed that ID@E-MSNs could accumulate tumor tissue and inhibit its growth and metastasis. Thus, tumor exosome-biomimetic nanoparticles indicate that a proof-of-concept could be concerned as a promising drug delivery system for efficient cancer combination therapy (shown in Figure 1).

## Results and Discussion

### Synthesis and Characterization of ID@E-MSN Nanoparticles

**FIGURE 2:**
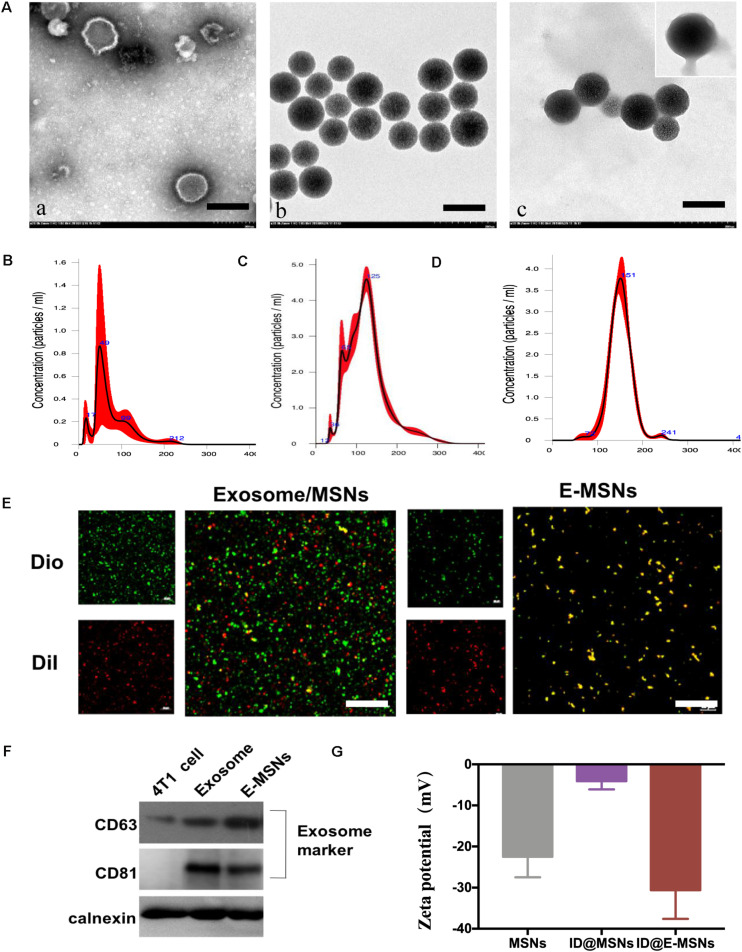
Synthesis and characterization. **(A)** TEM images of (a) 4T1 exosomes, (b) MSNs, and (c) E-MSNs, the membrane of E-MSNs with the average value of 20 nm. Scale bar: 100 nm. **(B–D)** The size distribution of 4T1 exosomes, MSNs, and E-MSNs was measured by NTA. **(E)** Confocal images of exosome membrane labeled by DiO dye (λ_em_ = 488 nm), DiI loaded in MSNs (λ_em_ = 560 nm), a mixture of exosome and MSNs, fused E-MSNs nanovesicles (Scale bar = 42 μm). **(F)** Western blot analysis of protein expression (CD63, CD81, calnexin) of 4T1 cells, 4T1 exosome, and E-MSNs nanovehicles. **(G)** Zeta potential (ζ) of MSNs, ID@MSNs, and ID@E-MSNs.

In this study, a biomimetic nanocarrier system was assembled based on 4T1 tumor exosome-modified MSNs for the co-loading of ICG and DOX, thus hoping to combine chemotherapy and photothermal therapy against breast cancer efficiently. The formulation of E-MSNs was composed of three processes: 4T1 exosomes were derived from the culture supernatants of 4T1 cells by ultracentrifugation; the mesoporous silica nanoparticles (MSNs) were synthetized under the guidance of previous methods; 4T1 exosomes were mixed with MSNs and then processed through extrusion, thus preparing E-MSNs. The typical morphological structure of these particles was observed by transmission electron microscope (TEM). The images of TEM (Figure 2A) revealed exosomes which had a typical morphology, MSNs particles displayed irregular morphology, and E-MSNs had 20 nm thick membrane appearing on the surface comparing with MSNs, confirming the presence of the membrane sheathed on MSNs in E-MSNs. In addition, the histogram of size distribution is shown in Figures 2B–D. NTA analysis displayed that the size of 4T1 exosomes was within 50–100 nm, MSNs and E-MSNs were 125 ± 15 nm and 150 ± 11 nm, in accordance with the result attained from TEM. To further prove that MSNs were coated with 4T1 exosomes membrane structure in E-MSNs, 3,3′-dioctadecyloxacarbocyanine perchlorate (DiO), a commonly used cell membrane fluorescent probe, was used to stain 4T1 exosomes, and 1,1′-Dioctadecyl-3,3,3′,3′-tetramethylindocarbocyanine perchlorate (DiI) was loaded in MSNs. What’s more, colocalization of green DiO fluorescence with red DiI fluorescence was observed in DiI@E-MSNs by confocal laser scanning microscopy (CLSM) (Figure 2E).

Apart from those, Western blot experiments further showed that similar to the whole cell lysates and the purified exosomes obtained by differential ultracentrifugation, exosome biomarkers CD63 and CD81 were also detected in E-MSNs (Figure 2F), confirming the presence of 4T1 exosomes in E-MSNs. In contrast to exosome biomarkers, calnexin, a protein located in the endoplasmic reticulum (ER), was only detected in whole cell lysates, but not in both E-MSNs and the purified exosomes, revealing the high purity of the exosomes sheathed on MSNs in E-MSNs.

E-MSNs was used as a drug carrier by co-loading ICG and DOX for combined therapy. ICG/DOX were loaded into MSNs and then ID@E-MSNs were also obtained by an extrusion method in a similar fashion to E-MSNs. In order to prove that E-MSNs successfully had loaded ICG and DOX, compared with the single MSNs, free ICG, and free DOX, ID@E-MSNs successfully detected two drugs with peaks of 488 and 780 nm, respectively (Supplementary Figure S1). Moreover, the zeta potential (ζ) values provided further evidence for the successful construction of nanoparticles co-loading drugs in each procedure, with a value of −20.5 ± 1.2 mV for MSNs, −5.8 ± 1.5 mV for ID@MSNs and −28.9 ± 3 mV for ID@E-MSNs (Figure 2G).

Meanwhile, ICG/DOX loading did not significantly change the size of E-MSNs. Moreover, the size of ID@E-MSNs remained almost constant even after incubating in PBS with or without 10% fetal bovine serum (FBS) for 7 days (Supplementary Figure S2). These results demonstrated that ID@E-MSNs were featured with excellent stability, which can be further applied for *in vitro* and *in vivo* study.

### Cellular Internalization of ID@E-MSNs

Cellular internalization plays a key role in therapy. To evaluate the cellular uptake efficiency of ID@MSNs, 4T1 cells were treated with free DOX+ICG, ID@MSNs and ID@E-MSNs. As shown in Supplementary Figure S3, the red fluorescence of DOX and the green fluorescence of ICG were observed in cells. After the 6 h incubation, compared with other treatments, ID@E-MSNs exhibited the highest fluorescence, indicating that exosome membrane coating enhanced the cellular uptake of ID@E-MSNs.

### Combination Therapy Effects of ID@E-MSNs *in vitro*

**FIGURE 3:**
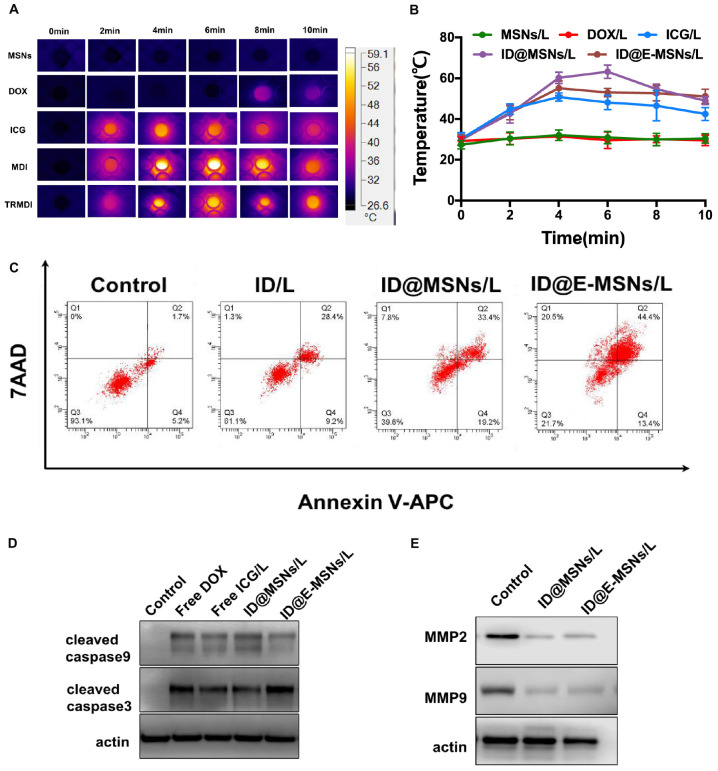
Combination therapy effects of ID@E-MSNs *in vitro*. **(A)** The IR thermal images of PBS, free DOX, free ICG, ID@MSNs, and ID@E-MSNs during laser irradiation for 10 min. **(B)** The temperature change curves of PBS, free DOX, free ICG, ID@MSNs, and ID@E-MSNs during laser irradiation. **(C)** The cell apoptosis quantified by the flow cytometry at 48 h after various treatments (Q1: live cells; Q2: early apoptotic cells; Q3: late apoptotic cells; Q4: dead cells). **(D)** The Western blot analysis of the protein expressions of cleaved caspase-9/-3 in 4T1 cells at 48 h after various treatments. **(E)** The Western blot analysis of the protein expressions of MMP2/9 in 4T1 cells at 48 h after various treatments.

To evaluate the photothermal efficiency of ID@E-MSNs *in vitro* by detecting the temperature changes in 10 min laser irradiation, the images of infrared thermal and curve of temperature were shown in Figures 3A,B. According to the result, the free ICG, ID@MSNs, ID@E-MSNs showed similar temperature changes, with the maximum temperature about 60°C, which was enough to kill tumor cells. These results indicated that ID@E-MSNs had the capacity of photothermal conversion equal to free ICG, and the exosome membrane coating exerted little impact on the ability to transfer light to heat, then to evaluate the release of DOX from ID@MSNs and ID@E-MSNs in different conditions (Supplementary Figure S4). The results showed the acidic condition and laser irradiation played a key role in stimulating the release of DOX from ID@E-MSNs. The cytotoxicity of free DOX, free ICG, and irradiation were evaluated by CCK-8 assay (Supplementary Figure S5). What’s more, the 808 nm laser at power densities of 1.0, 2.0, and 2.5 W/cm^2^ did not influence the growth of 4T1 during continuous irradiation for 10 min. Besides, the IC50 of free DOX was about 0.5 μg/mL, and that of free ICG and NIR irradiation was 2 μg/mL. ID@E-MSNs exhibited combined therapy efficiency under laser irradiation to 4T1 breast cancer cells. To evaluate the effect of synergistic cytotoxicity, clone formation assay of 4T1 was performed. The results showed that 808 nm laser irradiation induced cell death. Moreover, the effect of synergistic cytotoxicity was assessed by the flow cytometry (Figure 3C). After laser irradiation, the total rate of cell apoptosis induced by ID@MSNs was 60.4%, while that of ID@E-MSNs was 78.3%. The cytotoxicity of ID@MSNs was more significant than the other treatments. Altogether, ID@E-MSNs mediated combinatorial chemo-photothermal therapy which induced 4T1 cell apoptosis. In addition, apoptosis-associated proteins and metastases-associated proteins were also detected. As shown in Figures 3D,E, after laser irradiation, the caspase-9/-3 expressions level in the 4T1 treated with ID@MSNs and ID@E-MSNs were increasing obviously, while the MM2/MMP9 level was declining, demonstrating that these treatments had activated the signaling pathway.

### Evaluation of ID@E-MSNs *in vivo*

**FIGURE 4:**
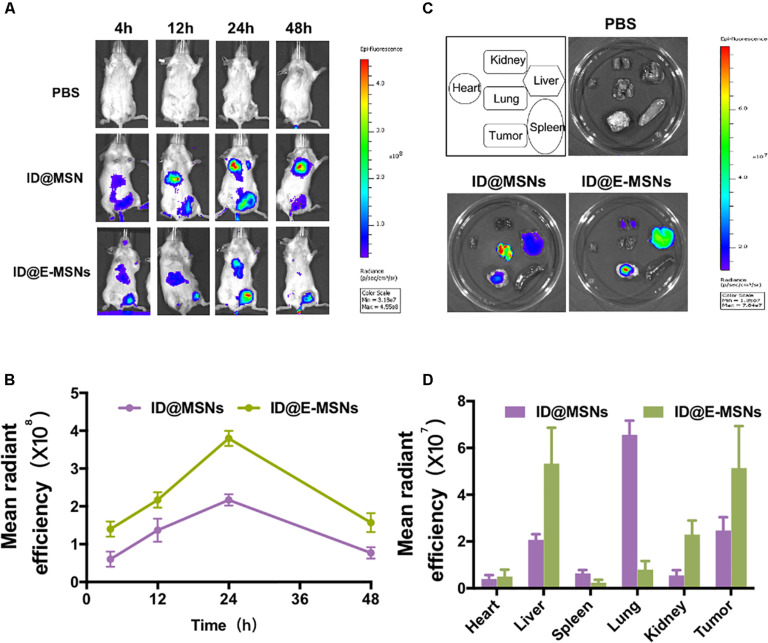
Distribution of ID@E-MSNs *in vivo*. **(A)** Imaging of 4T1 tumor-bearing mice at time points 4, 12, 24, 48 h after intravenous injection with ID@MSNs and ID@E-MSNs. Schematic diagram of experiment for 16 days of treatment. **(C)** Fluorescence images of organs harvest from 4T1 tumor-bearing mice were injected by ID@MSNs and ID@E-MSNs after 24 h. Schematic diagram of experiment for 16 days of treatment. **(B)** The fluorescence intensity of ID@MSNs and ID@E-MSNs *in vivo*. **(D)** The fluorescence intensity of ID@MSNs and ID@E-MSNs in organs.

According to the above results, the biodistribution of ID@E-MSNs was then taking a further investigation. The ID@MSNs and ID@E-MSNs were injected via tail veins of the 4T1 tumor-bearing mice to compare tumor accumulation capacity and photothermal capacity. As shown in Figures 4A,B, the fluorescent intensity of ID@E-MSNs gradually got enhanced at the tumor site during the first 24 h and were maintained for more than 24 h, which was always higher than that of the ID@MSNs group. These results showed that the tumor-targeting ability of ID@E-MSNs was from the modification of 4T1 exosome. Furthermore, the fluorescent intensity of tumors tissue injected by ID@E-MSNs was 3.1-fold higher than those injected by ID@MSNs (Figures 4C,D), proving the strong accumulation capacity of ID@E-MSNs. Nevertheless, the photothermal capacity of ID@E-MSNs and ID@MSNs was evaluated in Supplementary Figure S6. Upon NIR irradiation 24 h after injection, the tumor tissue administered by ID@E-MSN temperature gradually increased, reaching 50°C, which was powerful enough to kill tumor cells.

### Combination Therapy Effects of ID@E-MSNs *in vivo*

**FIGURE 5:**
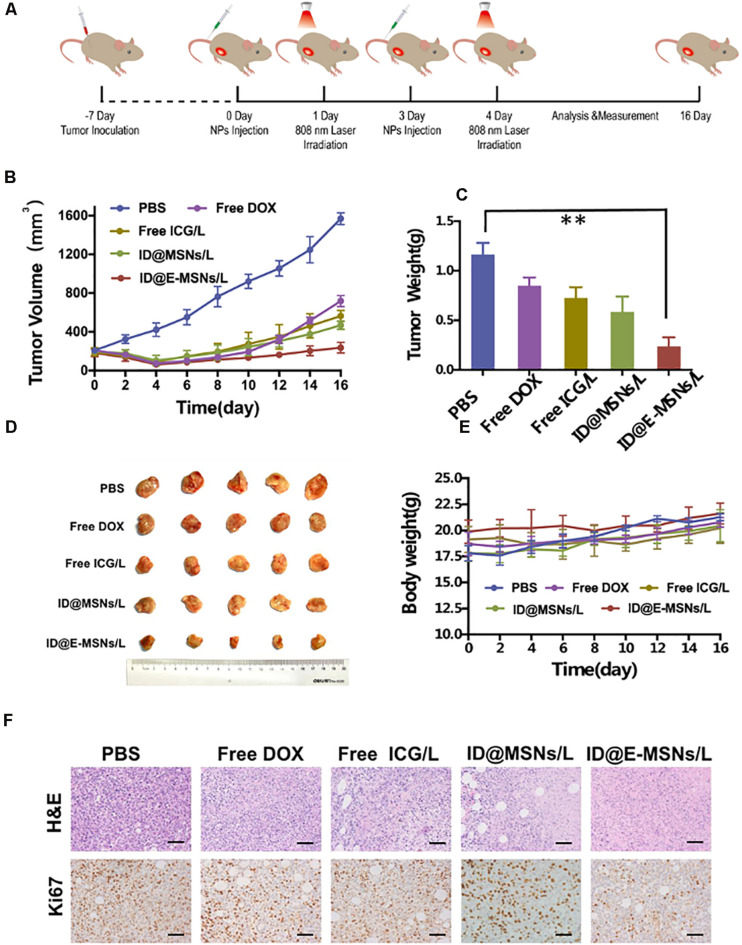
Combination therapy effects of ID@E-MSNs *in vivo*. **(A)** Schematic diagram of experiment for 16 days of treatment. **(B)** The curves of tumor volumes, *n* = 5. **(C)** The photograph of tumor, *n* = 5. **(D)** Tumor weight, *n* = 5. Data are characterized as mean ± *SD*, **p < 0.01. **(E)** The curves of body weight, *n* = 5. **(F)** HE and Ki67 staining of tumor tissues after 16 days of treatment, Scale bar = 1 mm.

To investigate the impact on the therapeutic effect and the inhibition of the metastasis of ID@E-MSNs, chemo-photothermal therapy schedule conducted in 4T1-tumor-bearing BALB/c mice is shown in Figure 5A. Tumor-bearing mice were administered with PBS, Free DOX, Free ICG+NIR laser, ID@MSNs+NIR laser, and ID@E-MSNs+NIR laser. In all treatment groups, ID@E-MSNs-based chemo-photothermal therapy obviously inhibited the growth of tumor after 16 days of treatment (Figures 5B,C), in accordance with the photograph of tumors (Figure 5D). Also, there were no significant alterations shown in the body weight of mice with the injected ID@E-MSNs and exposed to NIR laser (Figure 5E). In addition, compared with the other groups, tumor cell proliferation was also observed in the groups by the Ki67 assay (Figure 5F), in which the ability of proliferation was repressed by the treatment of ID@E-MSNs+NIR laser. Therefore, ID@E-MSNs represented a very effective chemo-photothermal nanomedicine for the preventing growth and metastasis of breast cancer.

**FIGURE 6:**
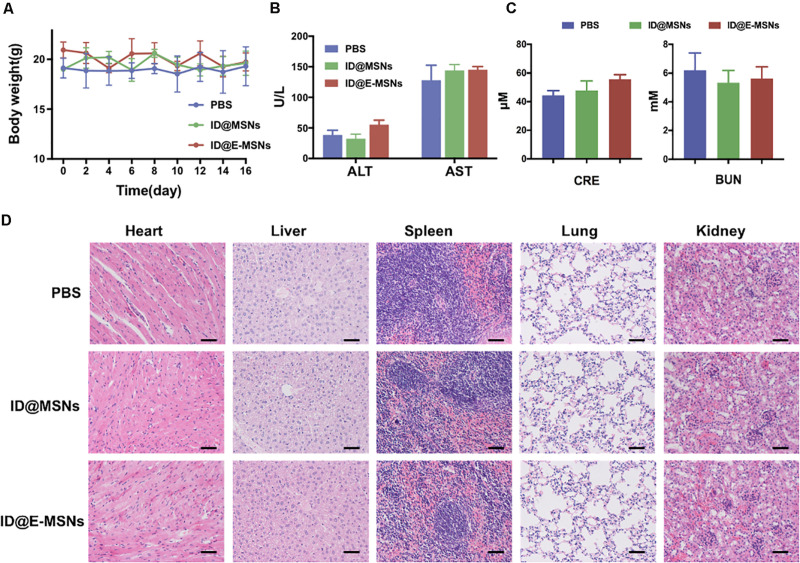
Biosafety assay verification of ID@E-MSNs. **(A)** Body weight curve of mice during 16 days of treatment. **(B)** Blood analysis of the levels of liver function markers ALT and AST. **(C)** Blood analysis of the levels of kidney function markers CRE and BUN. **(D)** H&E staining of major organs showing no obvious histology changes comparing with control, Scale bar = 1 mm.

To further evaluate the systematic toxicity of ID@MSNs and ID@E-MSNs *in vivo*, we injected the particles into healthy BALB/c mice by means of a tail-intravenous injection at a dosage of 20 mg/kg and harvested the blood at 24 h for biochemistry assay. As shown in Figure 6A, the weight of mice did not change significantly. The levels of liver function markers such as AST\ALT, and the kidney marker such as BUN\CRE, were all in the normal range (Figures 6B,C). Furthermore, in Figure 6D, compared with control, organs and tissues such as heart, liver, spleen, lung, and kidney had no obvious pathological change in ID@MSNs- and ID@E-MSNs-treated groups, which suggested that there was no evidence of inflammatory response caused by ID@E-MSNs. Altogether, ID@E-MSNs-mediated combinatorial chemo-photothermal therapy can be deemed as a safe drug carrier against tumors.

## Conclusion

In summary, we have successfully developed a biocompatible tumor exosome-sheathed MSNs-based drug delivery platform for targeted tumor chemo-photothermal therapy, in which ID@E-MSNs are constructed by combining 4T1 exosomes and ID@MSNs via an extruding method to an achieve enhanced combined therapy for cancer. Compared with free ICG/DOX, the drug loaded on MSNs and cloaked by 4T1 exosomes has shown superior performance in targeting, long-term retention, and favorable biocompatibility. These results mainly originate from the nature characteristics and the component of exosomes. Our study clearly demonstrates that exosome-biomimetic nanoparticles can be used in combined therapy, and this approach provides a new idea for developing natural drug carriers to improve the efficacy of anticancer therapy.

## Materials and Methods

### Materials

Doxorubicin (DOX), indocyanine green (ICG), bovine serum albumin (BSA), tetraethylorthosilicate (TEOS), and cetyltrimethylammonium bromide (CTAB) were obtained from Sigma-Aldrich (United States). 3,3′-Dioctadecyloxacarbocyanine perchlorate (Dio) and 1,1′-dioctadecyl-3,3,3′,3′-tetramethylindocarbocyanine perchlorate (DiI) were bought from Biyuntian (China). Polyvinylidene fluoride membranes (PVDF) were acquired from Millipore (United States). MTT and Ki67 reagents were provided by Yeasen Corporation (China). RPMI 1640 medium, FBS, penicillin, and streptomycin were provided by Hyclone (United States). You Ning Wei Corporation (China) offered the other reagents, including acrylamide, mouse anti-MMP2 (Santa Cruz, United States), mouse anti-MMP9 (Santa Cruz, United States), rabbit anti-calnexin (Santa Cruz, United States), rabbit anti-CD81 (ProteinTech, Chicago, IL, United States), and rabbit anti-CD63 (Abcam, Cambridge, Britain).

### Characterization

The particle size of exosomes and membrane-coated nanoparticles were measured by Nanosight3300 (Malvern, Britain). The morphology of the particle was observed by a Transmission Electron Microscope (Hitachi, Japan). The content of drugs and granules is calculated by UV-Vis absorption spectroscopy (BioTek, United States). The cells were observed by inverted fluorescence microscope and laser scanning confocal microscope (Leica, Germany). The 808 nm laser irradiation instrument was obtained from Changchun New Industries (China). Exosome-encapsulated nanoparticles were constructed by an Avanti mini extruder (Avanti Polar Lipids). Ultrapure water was prepared by the Millipore Milli-Q system (Merck, Germany).

### Preparation of MSNs and ID@MSNs

The MSNs were synthesized by a previous protocol (Cheng et al., 2018). Briefly, 0.25 g of CTAB was added into 25 mL of deionized water, 7 mL of absolute ethanol, and 70 μL of diethanolamine. The mixture was stirred at 70°C for 30 min and then quickly added 0.8 mL of TEOS. The action lasted for 1 h, then the template was removed to obtain the MSNs. After being freeze-dried, 10 mg of MSNs was weighted and mixed with 2 mg of DOX and 4 mg of ICG in 2 mL of deionized water, then stirred overnight at room temperature to prepare ID@MSNs. The ID@MSNs were centrifuged at 5000 rpm for 10 min to collect precipitate and gently washed with deionized water twice to eliminate free drug.

### Preparation of Purified 4T1 Exosome Vesicles

Firstly, 1 × 10^6^ 4T1 cells were seeded in 10 mL of 1640 supplemented with 10% no-nanovesicle FBS at standard condition for 48 h. The medium was then centrifuged at 5000 × *g* for 30 min, 10,000 × *g* for 30 min, and 100,000 × *g* for 1 h. The isolated exosomes were resuspended in cooling PBS. All the experiments were completed under 4°C. The preparation of 4T1 exosome vesicles used a previous method (Xin et al., 2017). In short, exosomes were sonicated at 5 W for 5 s, and then extruded with polycarbonate membrane to obtain exosomes vesicles. The protein of exosome vesicles was detected by Western blot.

### Fabrication of ID@E-MSNs Nanovehicles

ID@MSNs were embedded into 4T1 exosomes vehicles to fabricate the ID@E-MSNs nanovehicles by an extruding process. Briefly, 500 μL of exosomes (2 × 10^9^ particles/mL) were mixed with the equal particles of ID@MSNs in an Avanti mini extruder. Then the mixture was sonicated at 5 W for 5 s, and then extruded with polycarbonate membrane to obtain ID@E-MSNs. The protein of ID@E-MSNs was detected by Western blot, and the stability of ID@E-MSNs in PBS was tested for 7 days at room temperature.

### Cell Culture and Animals

Tumor cell 4T1 and mononuclear macrophage RAW264.7 cells bought from the American Type Culture Collection were cultured by a standard method (RPMI 1640 with 10% FBS and 1% penicillin and streptomycin at 37°C, 5% CO_2_); 18–20 g of BALB/c mice (female) were purchased from Beijing HFK Bioscience Co., Ltd. All of the animal experiments were compiled by relevant ethical regulations and were authorized by the Institutional Animal Care and Use Committee of Tianjin Medical University Cancer Institute.

### Cell Uptake Assay

1 × 10^6^ 4T1 cells and RAW264.7 cells were seeded into 10 cm dishes, respectively, supplemented with RIPM 1640 and 10% FBS at standard condition for 48 h. The cells were incubated with ID@MSNs and ID@E-MSNs for 4 h at the ICG concentrations of 10 μg/mL. After incubation, the free particles were washed three times by PBS, and the cells were stained with DAPI for 20 min for observation. Analysis was performed by a confocal microscope. Moreover, the uptakes of ID@MSNs and ID@E-MSNs in 4T1 cells and RAW264.7 were evaluated by the flow cytometry. In short, 1 × 10^6^ the cells were cultured in 6 cm dishes and incubated with ID@MSNs and ID@E-MSNs for 4 h at the ICG concentrations of 10 μg/mL. After incubation, the free particles were washed three times by PBS, and the cells were stained with DAPI for 20 min for observation. Analysis was performed by flow cytometry.

### Cell Safety Assay

5 × 10^3^ 4T1 cells were cultured into a 96-well plate for 24 h, then exposed to 808 nm laser irradiation at power density of 0.5, 1.5, and 2.5 W/cm^2^ for 10 min.

Subsequently, the cells were incubated for another 24 h. Then, the cells were processed by CCK8 assay to analyze the toxicity of irradiation, and the absorbance was detected by a microplate reader. The same method was used to evaluate the toxicity of dosing.

### Biodistribution Study of ID@E-MSNs in Tumor-Bearing Mice

To construct the tumor-bearing mice, 1 × 10^5^ 4T1 cells were implanted in female BALB/c mice. When the tumor volumes reached 150 mm^3^, the biodistribution studies of ID@E-MSNs were evaluated. The experiments were divided into two groups (five mice for each group): 100 μL of ID@MSNs and ID@E-MSNs (ICG concentration: 100 μg/mL) was injected via tail veins to the 4T1 tumor-bearing mice. Fluorescent images were acquired by IVIS Spectrum imaging systems at desired time points – 1, 4, 8, and 24 h pre-injection. Under the same condition, the excised tumor and organs such as heart, liver, spleen, lung, and kidney were also imaged by the IVIS fluorescent system.

### *In vivo* Anti-tumor Assay of ID@E-MSNs

Orthotropic 4T1 tumor-bearing mode were constructed for assessing efficacy of combined therapy *in vivo*. When the tumor volumes reached about ∼150 mm^3^, the mice were randomized into two groups. The mice were intravenously injected with: PBS, free ICG/DOX, ID@MSNs, and ID@E-MSNs at an ICG dosage of 2 mg/kg and DOX of 0.5 mg/kg via the tail vein, respectively. The amount of ICG was 2 mg/kg and DOX was 0.5 mg/kg; 808 nm laser treatment was performed next day. The body weight of mice was recorded every day after treatment, and the dimensions of tumor were measured every 3 days. At 16 days after treatments, the mice were euthanized. Then, the tumor tissues and organs, such as the heart, kidney, liver, spleen, and lung were collected for histopathological examination (H&E) or Ki67 staining. At 24 h post-injection, the levels of liver function markers such as AST\ALT and the kidney marker such as BUN\CRE were detected by kit.

### Statistical Analysis

All values represented mean ± *SD*. Statistical analyses between two paired groups using the Tukey comparative test (ANOVA) were carried out by the GraphPad Prism 5.0 software. *P* < 0.05 indicates statistically significant data.

## Data Availability Statement

The original contributions presented in the study are included in the article/Supplementary Material, further inquiries can be directed to the corresponding author/s.

## Ethics Statement

The animal study was reviewed and approved by the Institutional Animal Care and Use Committee of Tianjin Medical University Cancer Institute.

## Author Contributions

JC, WG, and RN designed the project. RT and ZW performed the experiments and analyzed data. HW interpreted the data and wrote the manuscript. All authors contributed to the article and approved the submitted version.

## Conflict of Interest

The authors declare that the research was conducted in the absence of any commercial or financial relationships that could be construed as a potential conflict of interest.
