# Supplementary material for: Tumor Exosome Mimicking Nanoparticles for Tumor Combinatorial Chemo-Photothermal Therapy
Source: Front Bioeng Biotechnol. 2020 Aug 31;8:1010. doi: 10.3389/fbioe.2020.01010 (PMC7487365; doi:10.3389/fbioe.2020.01010)
Supplement: Supplementary file 1 [file Image_1.pdf]

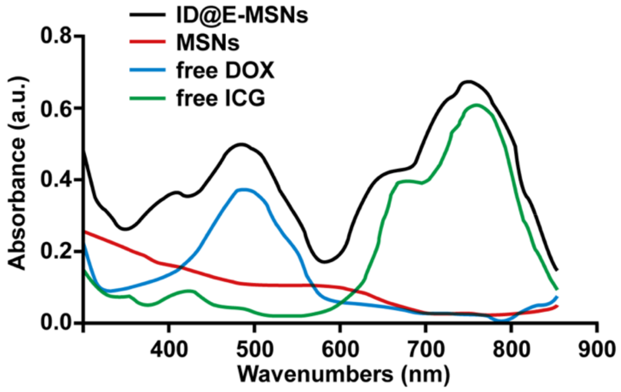

Supplementary Figure 1. UV–vis spectroscopy of ID@E-MSNs, free DOX, free ICG and MSNs.

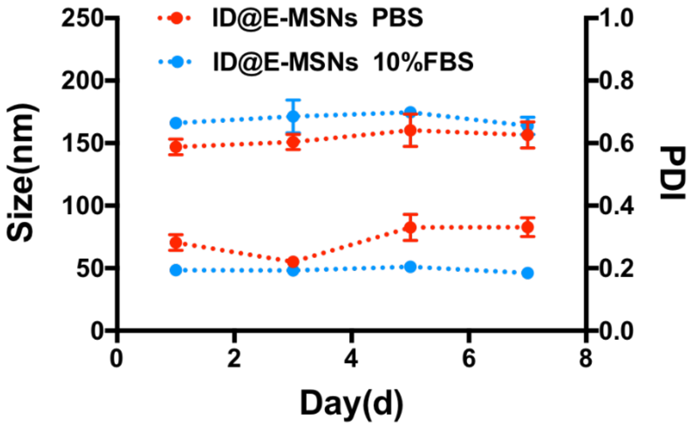

Supplementary Figure 2. The stability of ID@E-MSNs in PBS and 10% FBS during 7 days.

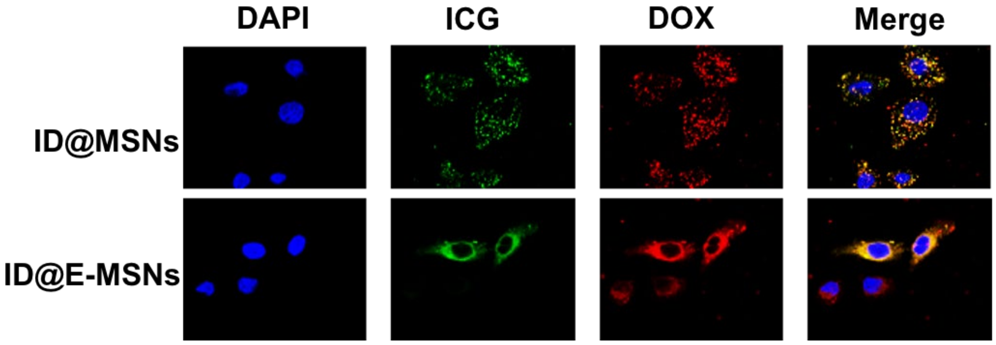

Supplementary Figure 3. The cellular uptake efficiency of ID@MSNs and ID@E-MSNs in 4T1 cells after 6 h incubation.

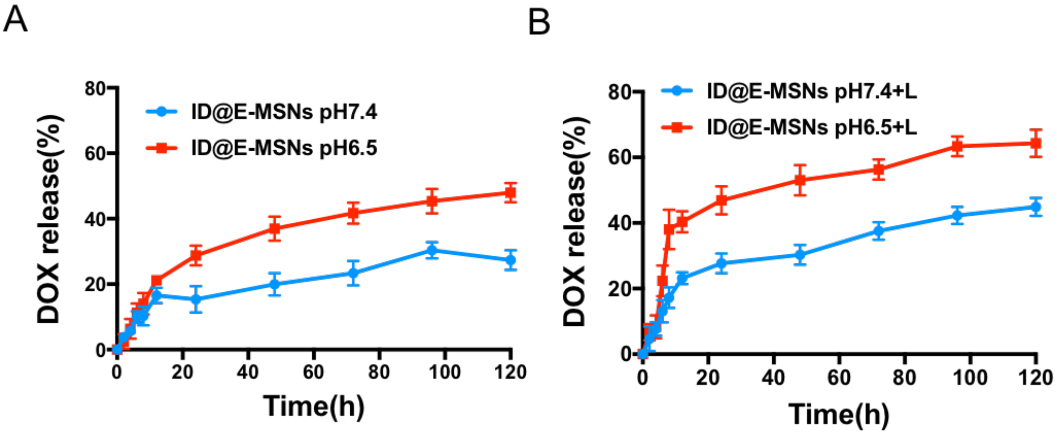

Supplementary Figure 4. The release of DOX from ID@E-MSNs **(A)** without laser irradiation. **(B)** with laser irradiation at different pH. In all experiments, the laser irradiation was carried out at 808 nm at power density of 2 W/cm^2.

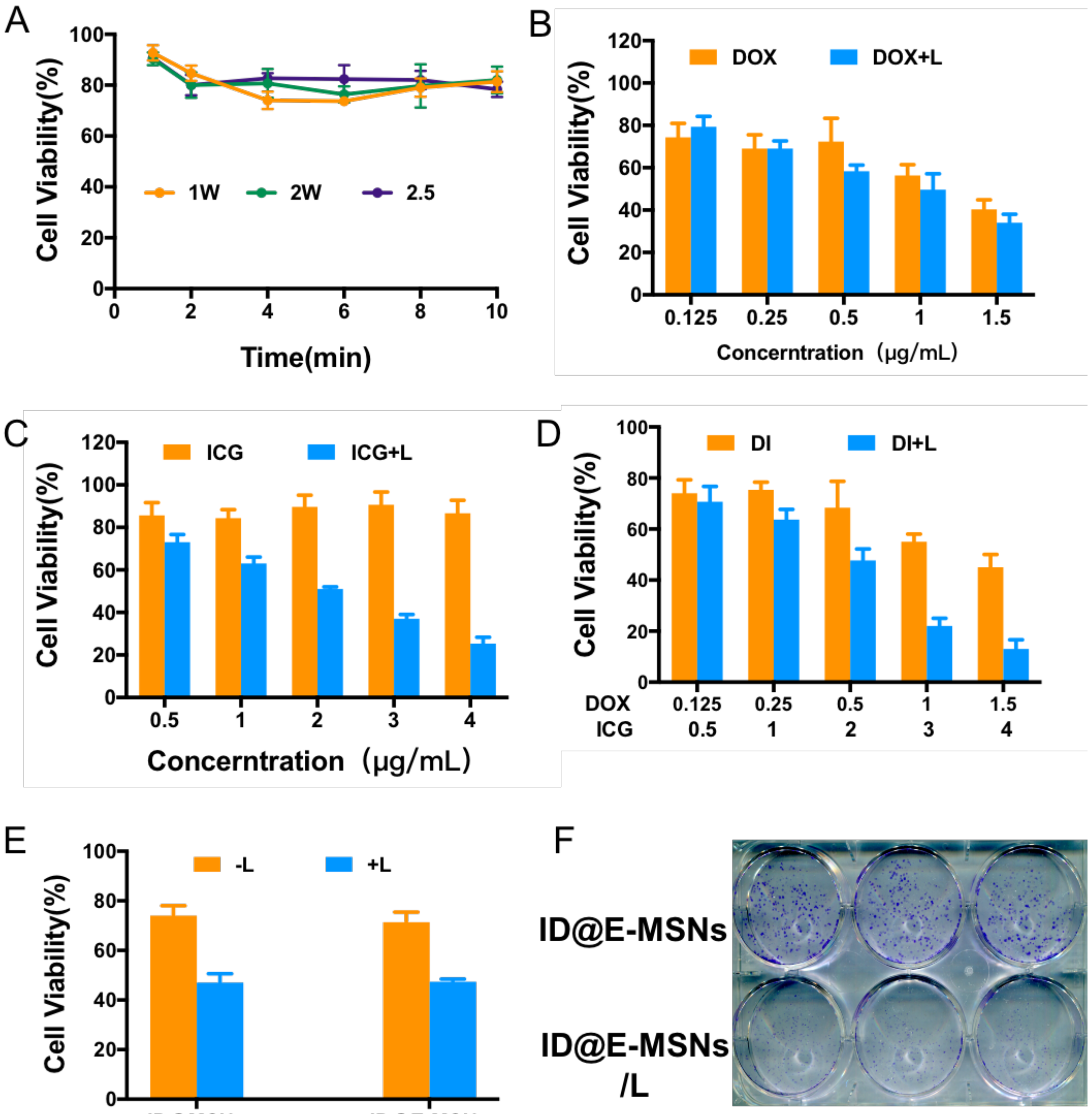

Supplementary Figure 5. *In vivo* the cytotoxicity of **(A)** Laser irradiation. **(B)** Free DOX. **(C)** Free ICG. **(D)** Free DOX+ICG. **(E)** ID@MSNs and ID@E-MSNs. **(F)** To evaluate the effect of synergistic cytotoxicity by clone formation assay.

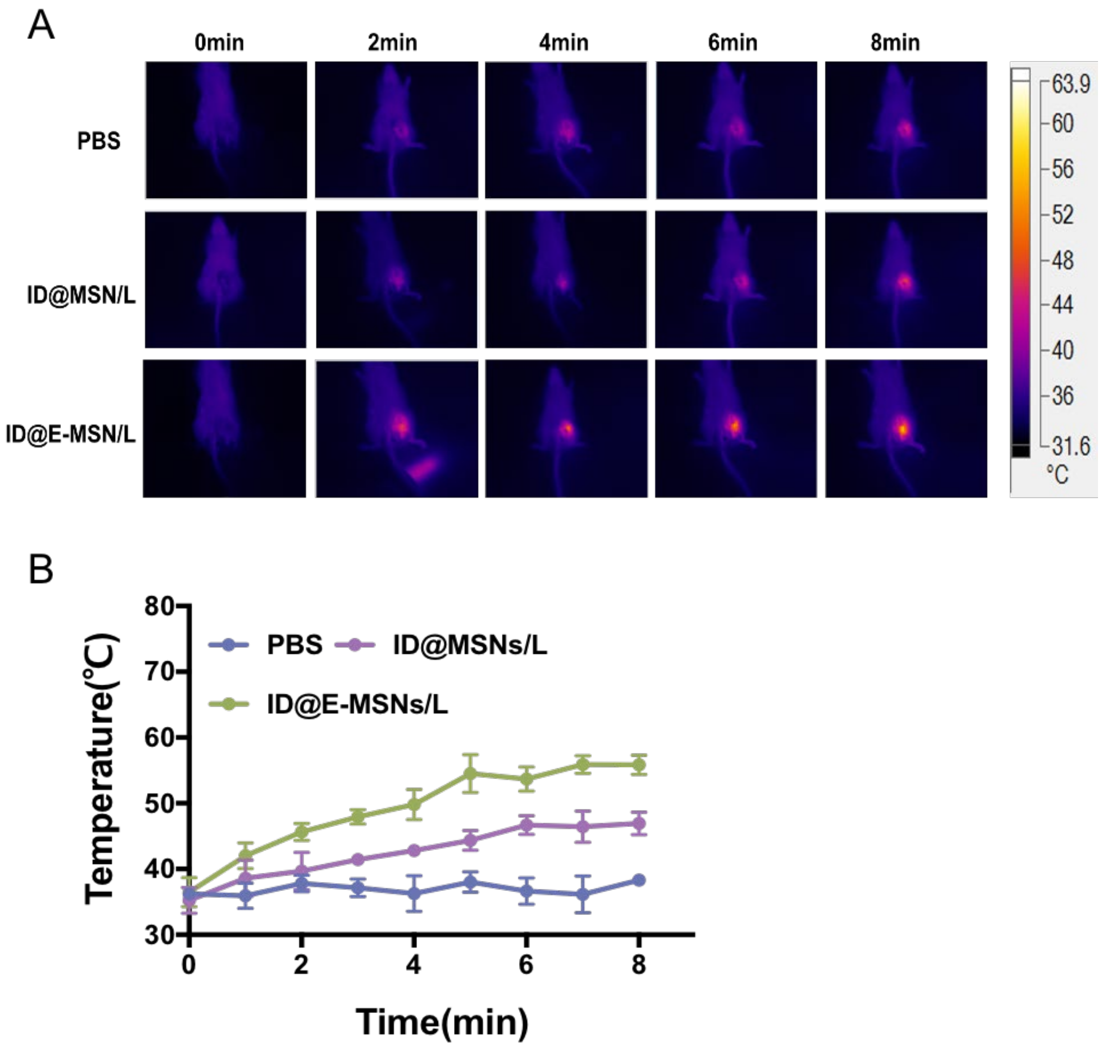

Supplementary Figure 6. *In vivo* photothermal efficacy of ID@E-MSNs. **(A)** The NIR thermal images of 4T1 tumor-bearing mice during laser irradiation after intravenous injection with PBS, ID@MSNs and ID@E-MSNs. **(B)** The temperature change curves of PBS, ID@MSNs, and ID@E-MSNs during laser irradiation.
